# Taxonomic and functional trait diversity of wild bees in different urban settings

**DOI:** 10.7717/peerj.3051

**Published:** 2017-03-07

**Authors:** Étienne Normandin, Nicolas J. Vereecken, Christopher M. Buddle, Valérie Fournier

**Affiliations:** 1 Centre de Recherche en Innovation sur les Végétaux, Université Laval, Québec, Canada; 2 Landscape Ecology & Plant Production Systems Unit, Université Libre de Bruxelles, Bruxelles, Belgique; 3 Department of Natural Resource Sciences, McGill University, Ste-Anne-de-Bellevue, Canada

**Keywords:** Urban ecology, Urban agriculture, Exotic species, Dominant species, Urbanization, Synanthropic species, Community ecology, Bee survey, Pollinator conservation, Biodiversity

## Abstract

Urbanization is one of the major anthropogenic processes contributing to local habitat loss and extirpation of numerous species, including wild bees, the most widespread pollinators. Little is known about the mechanisms through which urbanization impacts wild bee communities, or the types of urban green spaces that best promote their conservation in cities. The main objective of this study was to describe and compare wild bee community diversity, structure, and dynamics in two Canadian cities, Montreal and Quebec City. A second objective was to compare functional trait diversity among three habitat types (cemeteries, community gardens and urban parks) within each city. Bees were collected using pan traps and netting on the same 46 sites, multiple times, over the active season in 2012 and 2013. A total of 32,237 specimens were identified, representing 200 species and 6 families, including two new continental records, *Hylaeus communis* Nylander (1852) and *Anthidium florentinum* (Fabricius, 1775). Despite high community evenness, we found significant abundance of diverse species, including exotic ones. Spatio-temporal analysis showed higher stability in the most urbanized city (Montreal) but low nestedness of species assemblages among the three urban habitats in both cities. Our study demonstrates that cities are home to diverse communities of wild bees, but in turn affect bee community structure and dynamics. We also found that community gardens harbour high levels of functional trait diversity. Urban agriculture therefore contributes substantially to the provision of functionally diverse bee communities and possibly to urban pollination services.

## Introduction

Urbanization is pervasive worldwide, and dramatically modifies environments (Aronson et al., 2014; Shochat et al., 2010; McDonnell & Hahs, 2008; McKinney, 2002). Global urban sprawl and infrastructure development has increased considerably in the past 100 years, and this trend will continue well in the 21st century, with an estimated 66% of the world’s population living in cities by 2050 (United Nations Department of Economic, Social Affairs Population Division, 2014). The ongoing expansion of paved surfaces, buildings, devegetated lands and human activities will amplify habitat loss, a leading cause of species extinction and shrinking biodiversity (McKinney, 2002). However, urbanization does not simply extirpate species, but it may also cause shifts in community structure through the conversion of one type of habitat into another (Shochat et al., 2010). It is therefore a pressing priority to gain insights into the full range of these effects, especially on species that contribute to the provision of ecosystem services, such as wild bees, so important for crop pollination (Kleijn et al., 2015; Gallai et al., 2009; Kearns, Inouye & Waser, 1998).

Several studies have shown that wild bees are thriving in cities (e.g., Sirohi et al., 2015; Fortel et al., 2014; Banaszak-Cibicka & Zmihorski, 2012; Bates et al., 2011; Fetridge, Ascher & Langellotto, 2008; Matteson, Ascher & Langellotto, 2008). They may be taking advantage of the fine scale heterogeneity (Cadenasso, Pickett & Schwarz, 2007) and combination of human variables and vegetation features occurring in urban environments (Matteson, Grace & Minor, 2013; Lowenstein et al., 2014). Parks and gardens can host a wide variety of ornamental and exotic plant species (Thompson et al., 2003; Frankie et al., 2005) among which pollen generalist (i.e., polylectic) bees with broad tolerance can prevail over specialist (i.e., oligolectic) species and prosper (Cane, 2005; Eremeeva & Sushchev, 2005). However, we still know little about the effect of different kinds of urban green spaces on bee community structure, which precludes gaining insight into the relationship between cities and the development of conservation strategies targeting urban wild bees. The dominance of some species and the presence of exotic ones can shape community structure (Shochat et al., 2010; McKinney, 2008), but few studies (Matteson, Ascher & Langellotto, 2008; Cane, 2005) have addressed how dominant species affect the overall structure of entire bee communities in urban environments. When a species becomes dominant, it can decrease community evenness, a key component of species diversity (Shochat et al., 2010; Marussich & Faeth, 2009; Niemelä & Kotze, 2009). This phenomenon may be driven by exotic species that are typically introduced to cities through human activities (McKinney, 2006). These effects of urbanization are important to investigate, but quantifying them requires monitoring and establishing benchmarks of the diversity of urban bees in multiple cities.

Urban green spaces can play an important role in the conservation and promotion of biodiversity in cities, and humans and wildlife can benefit from the same types of green space (Middle et al., 2014; Goddard, Dougill & Benton, 2009). Several studies have monitored bees in urban, structurally different green spaces, such as community gardens (Matteson, Ascher & Langellotto, 2008), cemeteries (Bates et al., 2011), parks (McFrederick & LeBuhn, 2006), and, comparatively, in all three of these together (Sirohi et al., 2015; Andersson, Barthel & Ahrné, 2007). Urban agriculture in community gardens has been found to offer multiple benefits, since it improves public health values (Fuller et al., 2007), and provides opportunities education (Graham et al., 2005) and food production of particular interest for low-income residents (Van Leeuwen, Nijkamp & De Noronha Vaz, 2010), as well as abundant floral resources to pollinators (Matteson & Langellotto, 2010). Community gardens provide structurally complex and taxonomically diverse communities of wild and cultivated flowering plants, which are good predictors of insect abundance and diversity (Smith et al., 2006). Despite these benefits, community gardens rarely receive the same level of attention from planners as do urban parks (Lawson, 2004). Although these gardens are visited by many wild bee species (Frankie et al., 2009; Fetridge, Ascher & Langellotto, 2008; Matteson, Ascher & Langellotto, 2008), research is needed to evaluate the contribution of community gardens to bee diversity in relation to other green spaces.

To compare urban habitats, the concept of functional diversity can provide important insights into community response, and bring more value than just focusing on taxonomic diversity (e.g., species richness) (Cadotte, Carscadden & Mirotchnick, 2011). By combining life history, morphological and ecological traits, functional diversity is considered an important component of diversity because it influences many aspects of ecosystem functioning (Tilman, 2001). In this study, we focused on a series of life-history traits used as descriptors of how wild bee species interact with their environment and each other (Violle et al., 2007). Such life-history traits include pollen specialization, seasonal activity, trophic position (i.e., host, cleptoparasite, or social parasite), nesting behaviour, tongue length and body size. They can be compiled for each species and assessed by comparing the range and distribution of trait values within and among communities (Cadotte et al., 2015; Díaz, Noy-Meir & Cabido, 2001; Petchey & Gaston, 2006). Previous studies (e.g., Fontaine et al., 2005; Hoehn et al., 2008; Martins, Gonzalez & Lechowicz, 2015) have shown a link between trait diversity of wild bees and ecosystem function (i.e., improved pollination and therefore plant reproduction) through a mechanism described as interactive niche complementarity (Albrecht et al., 2012; Fründ et al., 2013). This approach can be used to describe the total variation in multiple traits across all species within communities of wild bees as a simple proxy for their “functional diversity” in the context of the pollination of wild and cultivated plants in urban green spaces. While urbanization has been shown to be associated with the loss of functional diversity in many taxa (e.g., Flynn et al., 2009; Pauw & Louw, 2012), urban habitats with higher functional diversity may be more beneficial than sites that simply host more species. This may be particularly true for bees, which provide pollination services and are likely to be affected differently according to the type of urban habitats they visit.

In the last 25 years, urban sprawl in Montreal and Quebec City has increased dramatically and has outpaced urban planning strategies (Nazarnia, Schwick & Jaeger, 2016). Because urbanization can have detrimental effects on wildlife (e.g., Soulsbury & Piran, 2016), the need to focus on important insects, such as wild bees, is imperative. Here, we conducted standardized wild bee sampling in these two Canadian cities, multiple times over the active (snow-free) season, over a period of two years. Our first objective was to describe and compare the diversity, structure and dynamics of bee communities between the two cities. We used broad metrics to describe the communities, including partitioning of beta diversity, occurrence of synanthropic, dominant and exotic species, species evenness in communities, and spatio-temporal stability. Since Montreal has a higher population density than Quebec City (Nazarnia, Schwick & Jaeger, 2016), we predict its wild bee community structure and dynamics should be different. We also predict that the species assemblages (community composition) will be nested in the three habitats sampled. Our second objective was to compare the functional trait diversity of bees among three urban habitats in each city: cemeteries, community gardens and urban parks. We expect functional trait diversity to provide insights into the significance of these three green spaces and help evaluate their potential for the conservation of functionally diverse bee communities in urban settings. We predict that urban agriculture in community gardens contributes substantially to wild bee diversity in cities.

## Material and Methods

### Study design

The study was conducted in Montreal (45°30′04″N: 73°39′22″O) and Quebec City (46°48′08″N: 71°15′50″O), both located in the southern part of the province of Quebec, Canada, 230 km apart. Montreal is the second largest city in Canada, with a population of 1.6 million (Statistics Canada, 2014) (4 million in the greater metropolitan area). Quebec City has a population of 799,632 (Statistics Canada, 2014). Wild bees were collected in three types of urban green spaces on the same 46 sites in 2012 and 2013 (see sampling protocol below and Table 1 for the complete list of sites). In Montreal, 10 community gardens, nine cemeteries and five urban parks were sampled, while 10 community gardens, seven cemeteries and five urban parks were sampled in Quebec City.

The urban parks chosen for this study are preserved areas with mature forests. These sites are not exclusively recreational parks such as those common in residential neighbourhoods. They cover a relatively large area (see Table 1), and are characterised by a combination of mostly unmanaged forested areas and open areas in vegetational succession or extensive lawns for recreational use. Cemeteries occupy the next-largest areas among our study sites (see Table 1), and are characterised by a high proportion of managed lawns with sometimes forested or treed areas. Management of cemeteries is often guided by the aim of creating a peaceful, and attractive environment for visitors. These sites consequently include flowerbeds, native and exotic trees and natural or unmanaged vegetation growing along their borders. Community gardens vary in size (Table 1) and contain numerous garden plots used for growing herbs, vegetables or fruits; flowerbeds are common along their borders. Minimal and maximal distances between sites were 305 m and 41 km, respectively.

**Table 1:** List of the sites surveyed for bees in Montreal and Quebec City with their abbreviation, type, size and coordinates.

Site name	Abbreviation	Green space type	City	Size (m^2^)	Latitude, longitude	Longitude
Shaare Zion	CBS	Cemetery	Montreal	16,483	45,5503	−73,6557
Urgel Bourgie	CC	Cemetery	Montreal	62,100	45,5095	−73,6647
Hawthorn-Dale	CHD	Cemetery	Montreal	86,000	45,6838	−73,5069
Lachine	CL	Cemetery	Montreal	50,735	45,4435	−73,6840
Lakeview	CLV	Cemetery	Montreal	147,000	45,4417	−73,8373
La Présentation	CP	Cemetery	Montreal	13,005	45,4434	−73,7366
Ste-Geneviève	CSG	Cemetery	Montreal	44,800	45,4590	−73,9204
St-Laurent	CSL	Cemetery	Montreal	16,182	45,5155	−73,6714
De La Visitation	CV	Cemetery	Montreal	6,200	45,5674	−73,6591
Alexis-Nihon	JAN	Community garden	Montreal	1,050	45,4971	−73,6910
Étienne-Desmarteaux	JED	Community garden	Montreal	5,149	45,5581	−73,5782
Hartenstein	JH	Community garden	Montreal	567	45,5581	−73,5782
Laurendeau	JL	Community garden	Montreal	2,320	45,6036	−73,5702
Prieur	JP	Community garden	Montreal	4,221	45,5778	−73,6489
Père-Marquette	JPM	Community garden	Montreal	3,180	45,5399	73.595690
Roseraie	JR	Community garden	Montreal	2,117	45,5923	−73,5544
Rosemont-Églantier	JRE	Community garden	Montreal	7,514	45,5654	−73,5685
Sherbrooke	JS	Community garden	Montreal	960	45,4447	−73,6679
Sainte-Marthe	JSM	Community garden	Montreal	3,050	45,6386	−73,5981
Bois-de-Liesse	PBL	Nature park	Montreal	1,716	45,5007	−73,7647
Boucherville island	PIB	Nature park	Montreal	3,024	45,5885	−73,4854
Pointe-aux-Prairies Heritage	PPPH	Nature park	Montreal	5,900	45,6815	−73,5068
Pointe-aux-Prairies Marais	PPPM	Nature park	Montreal	9,211	45,6881	−73,5237
De La Visitation	PV	Nature park	Montreal	3,500	45,5808	−73,6561
Cap-Rouge	CCR	Cemetery	Quebec	6,770	46,7504	−71,3453
Notre-Dame-de-Belmont	CDB	Cemetery	Quebec	92,328	46,7903	−71,2787
Mount Hermont	CMH	Cemetery	Quebec	92,127	46,7788	−71,2469
Notre-Dame-de-Foy	CNDF	Cemetery	Quebec	7,051	46,7778	−71,3031
Saint-Augustin	CSA	Cemetery	Quebec	9,988	46,7433	−71,4580
Saint-Charles	CSC	Cemetery	Quebec	117,697	46,8087	−71,2725
Saint-Patrick	CSP	Cemetery	Quebec	52,611	46,7845	−71,2408
Duberger	JD	Community garden	Quebec	1,856	46,8115	−71,2918
Le Marmottier	JLM	Community garden	Quebec	2,845	46,7935	−71,2419
Louis-Riel	JLR	Community garden	Quebec	3,907	46,7650	−71,3047
Marchand	JM	Community garden	Quebec	1,510	46,8372	71.241248
Mont-Lilas	JML	Community garden	Quebec	8,316	46,8612	−71,1966
Parc des Sables	JPS	Community garden	Quebec	1,545	46,8214	−71,2221
Pointe Sainte-Foy	JPSF	Community garden	Quebec	9,046	46,7660	−71,3299
Sapinière Dorion	JSD	Community garden	Quebec	2,921	46,8445	−71,2403
Université Laval	JUL	Community garden	Quebec	7,131	46,7778	−71,2829
Versant Nord	JVN	Community garden	Quebec	3,593	46,7803	−71,3206
Cartier-Bréboeuf	PCB	Nature park	Quebec	28,503	46,8250	−71,2403
Bois-de-Coulonge	PBC	Nature park	Quebec	143,779	46,7897	−71,2382
Domaine Cataraqui	PDC	Nature park	Quebec	38,076	46,7750	−71,2530
Base de Plein air Sainte-Foy	PPASF	Nature park	Quebec	52,313	46,7911	−71,3283
Plage Jacques-Cartier	PPJC	Nature park	Quebec	22,584	46,7506	−71,3138

### Wild bee sampling

Wild bees were sampled at each site every two weeks using coloured pan traps and sweep-nets. The combination of methods is crucial, to properly assess bee communities (Westphal et al., 2008; Nielsen et al., 2011). Pan trapping is a standard and passive method that takes advantage of the insect’s attraction to certain colours. Plastic bowls with a capacity of 400 ml were used to construct pan traps. Originally white, one-third were spray-painted fluorescent blue and another third yellow (with Krylon^®^ paint); bowls were then grouped in threes, one each in white, yellow and blue. Bowls were raised to the average height of the herbaceous vegetation on a 60 cm long wooden stick. Bowls were filled with soapy water (5 drops of concentrated dish detergent per 1 L water) every two weeks during the sampling season. Pan traps were set up on all sites in a single day in one of the two cities, and were retrieved 48 h later all at once. As per Matteson, Ascher & Langellotto (2008), we used a ratio of pan traps per sampling area because the sites were highly variable in size. We installed one group of 3 bowls per 1,000 m^2^ with a maximum of 45 pan traps per site, for feasibility. In Montreal, we installed a total of 481 pan traps, and in Quebec City, 550.Whenever possible, pan traps were aligned along a single transect, 1–2 m apart. On some of the sites, especially in cemeteries, pan traps could not be placed on the highly managed lawns, and were therefore placed along borders. Additional sweep-netting is considered essential to this type of sampling, in order to collect wild bees from flowers as well as species that may be less attracted to pan traps (Westphal et al., 2008). Consequently, after every pan trapping period on each site (i.e., every two weeks), on sunny days, 10 min of active collection with a sweep net was conducted (45 cm diameter). Sweep netting was performed on open flowers and targeted bees. For both cities, the 2012 sampling began the first week of June and ended at the end of September; in 2013, sampling began the first week of May and also ended at the end of September. Overall, sampling (pan trap + sweep netting combined) was performed on every site (46 sites) 8 times/year, totalling 736 samples (46 sites × 8 samplings/site × 2 years). We received permission to collect insects in Montreal parks from the management of Montreal’s large parks and from the director of the Boucherville Islands national park. Authorizations to collect at the other sites (community gardens, cemeteries and Quebec city’s parks) were given verbally and are listed under the Field Study Permissions.

Collected specimens were stored, washed, processed, pinned and identified to the lowest taxonomic level using several keys (Mitchell, 1962; Michener, 2007; Gibbs, 2010; Gibbs et al., 2012) and the assistance of expert taxonomists (see ‘Acknowledgements’). The first author of this study identified nearly all specimens. Voucher specimens were deposited at Laval University (Fournier laboratory) and in the Lyman Entomological Museum at McGill University.

## Data Analyses

All analyses were performed using pooled data obtained by both sampling techniques (i.e., pan traps and netting) because these techniques can be considered to be complementary (Westphal et al., 2008). Sampling periods were also pooled, except for the abundance LSD test (ANOVA) comparing the three urban habitats.

### Species accumulation curves

We first used pooled data from all urban habitats, sampling years and sampling techniques to calculate species accumulation curves with the “vegan” package (Oksanen et al., 2013) in order to assess differences in species diversity between Montreal and Quebec City. We calculated the mean species accumulation curves and their associated standard deviation from *n* = 999 random permutations of the data, or subsampling without replacement (Gotellli & Colwell, 2001). We then calculated the observed species accumulation curves and total expected species richness (or the number of unobserved species) using a bootstrapping procedure with 999 random reorganizations of sampling order. Total expected species richness was assessed using Chao (Chao, 1984), Jack1 (First order jackknife), Jack2 (Second order jackknife) and Bootstrap estimators (Smith & Van Belle, 1984; Chao, 1987; Palmer, 1990; Colwell & Coddington, 1994; Walther & Morand, 1998). To compare diversity across habitats, we plotted species accumulation curves for each habitat within each city with the “vegan” package in R (Oksanen et al., 2013) using 999 random permutations.

To illustrate the number of bee species recorded in each habitat type (i.e., their original contribution in terms of species) for each city (Montreal and Quebec City), as well as the shared species (e.g. the number of species collected jointly in two or more habitats), we created a Venn diagram for each city with overlapping ellipses using the “VennDiagram” package (version 1.6.5) in R (Chen & Boutros, 2011). To describe rank-abundance distributions specific to our datasets for Montreal and Quebec City, we first plotted rank abundance–dominance (RAD) curves (1 for Montreal, 1 for Quebec City) (Whittaker, 1965) with the biodiversityR package in Rstudio. The data allowed us to determine which species could be considered abundant or dominant. All species that ranked higher than the curve plateau were considered “abundant” species. If a species represented more than 50% of the total abundance at a site with the two sampling years pooled, it was considered “dominant.”

### ANOVA and Welch’s *t*-test

To compare the abundance of exotic and dominant or abundant species between the two cities, the Welch two-sample *T* test was used. To compare abundance among the three habitats (park, cemetery and community garden), Fisher’s least significant test (ANOVA) was used. Abundance per pan trap was calculated first because of the pan trap ratio used (1 triplet per 1,000 m^2^). Then, four LSD tests were performed in R for each city and each sampling technique (pan trap and netting). For the Quebec City pan trap test, a LOG transformation was performed to correct variance homogeneity. For the netting technique, a square root transformation was performed for both cities.

### Heat map graphic

We plotted heat maps with the pheatmap package in R (Kolde, 2013) to visually highlight the differences in wild bee abundance among sites, particularly the extreme values (“outliers”) recorded for a few locally superabundant species.

### Analysis of species evenness among sites and habitats

To quantify how equal the communities of wild bees were numerically across sites and habitats, we computed Pielou’s evenness index (*J*′), a measure of diversity based on the ratio between the Shannon’s diversity index (*H*′) and the natural logarithm of species richness for each site. Values of Pielou’s index *J*′ are constrained between 0 and 1, with values close to 1 indicating that all recorded species are close in numbers locally (Pielou, 1975; Smith & Wilson, 1996). We computed the index for each study site using the “vegan” package in R (Oksanen et al., 2013).

### Inter-annual variation in bee community structure

To assess fluctuations in bee communities across sites, habitats and years (2012 vs. 2013), we first used the “pvclust” package in R (Suzuki & Shimodaira, 2006) to perform separate hierarchical clustering analyses with bootstrap resampling for each city and year, after eliminating the minority of study sites that did not have a corresponding dataset for the alternate year. We then linked the dendrograms obtained using the “dendextend” package (Galili, 2014) to produce a “tanglegram” that allowed us to test the degree of congruence between clustering analyses for each year through a set of metrics characterizing the quality of the dendrogram alignments and the occurrence of nested clusters. We first computed the entanglement index to characterize the quality of the alignment of the two trees in the “tanglegram” layout. Its values range between 0 and 1, with near 0 values meaning that the two trees are identical. We then computed Baker’s ɣ index (Baker, 1974) as a measure of association (similarity) between two dendrograms. Its values range between −1 and 1, with near 0 values meaning that the two trees are not statistically similar. Finally, we computed the cophenetic correlation coefficient (CCC) (Sokal & Rohlf, 1962), defined as the correlation between two cophenetic distance matrices of two trees, or how faithfully a dendrogram preserves the pairwise distances between the original unmodeled data points. The value can range between −1 and 1, with near 0 values meaning that the two trees are not statistically similar and positive values indicating a higher degree of similarity between the cophenetic distance matrices of the two trees (Galili, 2014).

### Partitioning of beta diversity among habitats

To estimate the multiple-site variation in species composition among habitats for each city, taking into account the identity of all species, we computed beta diversity metrics. We used the “betapart” package (Baselga & Orme, 2012), with which it is possible to partition the Sørensen index of beta diversity (*β*sor, a measure of total dissimilarity) into its two major components, (i) species replacement (i.e., “true” species turnover: *β*sim) and (ii) species loss/or gain (i.e., nestedness: *β*nes) according to *β*sor = *β*sim + *β*nes.

The Sørensen index (*β*sor) ranges from 0 (identical species assemblages) to 1 (different species assemblages). Using this approach with our dataset allowed testing (i) differences in the values of total dissimilarity (*β*sor) between Montreal and Quebec City, but also (ii) the relative contribution of species turnover (*β*sim) and nestedness-resultant dissimilarity (*β*nes) in each city.

### Functional trait diversity partitioning among habitats

We compiled ecological, life-history and morphological traits for all species recorded in this study. We chose six variables from a list of 30 variables of functional categorical traits, selected to include diverse attributes of wild bee ecology that are known to influence their functional role in an assemblage (Violle et al., 2007). The variables that concerned our species were: pollen specialization (oligolectic, polylectic), pollen transportation (accidental, leg and body, corbiculae, legs only, underside, crop), tongue length (short, medium, long), seasonal activity (spring, spring/summer), sociality (solitary, cleptoparasite, eusocial, communal), nesting behaviour (ground, cleptoparasite cavity below ground, cavity above ground, social parasite, carder), and inter-tegular distance. The inter-tegular distance (ITD) values, a good proxy for body size (Cane, 1987) and bee foraging range (Greenleaf et al., 2007; Zurbuchen et al., 2010 and references therein), were measured on five female specimens of each species with a digital calliper (precision = 0.01 mm). All of these trait attributes were allocated based on the primary literature (Ascher & Pickering, 2014), and on researcher expertise when published information for a particular species was unavailable (see also Moretti et al. (2009), who used the same approach).

The functional trait database was first used to construct a functional rarefaction curve based on species abundances, following the framework developed recently by Ricotta et al. (2012). This approach allowed us to investigate differences in functional diversity among habitats by computing Rao’s average quadratic functional diversity (${\overline{Q}}_{(M)}$) over all possible combinations of sampling plots (*M*), and by plotting ${\overline{Q}}_{(M)}$ as a function of *M*. This analysis also allowed us to test the relative contribution of each habitat type to the maintenance of the functional diversity of wild bees in each city.

All statistical analyses were performed with RStudio (2014) version 0.99.489.

## Results

**Figure 1:**
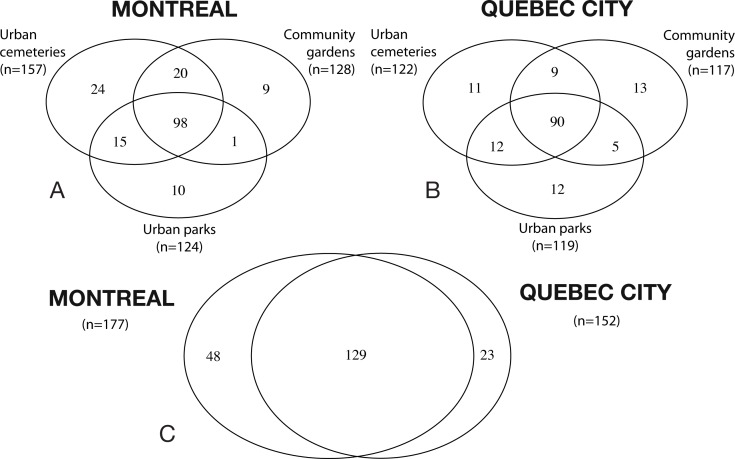
Venn diagrams illustrating species richness in Montreal and Quebec City. (A and B) Shows the species richness in the three urban habitats (cemeteries, community gardens and urban parks). (C) shows the overlapping of species richness between both cities.

A total of 32,237 specimens representing 200 species and six bee families (Andrenidae, Apidae, Colletidae, Halictidae, Megachilidae and Melittidae) were collected in 2012 and 2013. The Halictidae was the most abundant and diverse group, with 60 species (56% of the total specimens captured), followed by the Andrenidae, with 53 species (9%), the Megachilidae with 38 species (8%) and the Apidae, with 36 species (20%). A total of 24,233 bees and 177 species were collected in Montreal and 8,024 bees and 152 species for Quebec City. The mean number of bee per pan trap in Montreal (27.56 ± 3.35 (SE)) was significantly higher (*t*_1,45_ =  − 5.481, *P* < 0.001) than for Quebec City (8.27 ± 1.08 (SE)). The mean number of specimens collected by netting was also significantly higher (*t*_1,45_ = 4.895, *P* < 0.001) for Montreal (83.98 ± 11.48 (SE)) than Quebec City (27.80 ± 4.34 (SE)). The Venn diagrams (Fig. 1) show that 48 species are found only in Montreal, 23 species only in Quebec City and the cities share 129 species. The number of oligolectic (specialist) species was similar between the two cities, with 21 species in Montreal and 19 in Quebec City. Two new exotic species for North America, both originating from Western Europe, were recorded during this study: *Hylaeus communis* Nylander (1852) and *Anthidium florentinum* (Fabricius, 1775). In Montreal, 17 exotic bee species were recorded, accounting for 14% (3,477 individuals) of the total abundance. In Quebec City, 13 exotic species were recorded, accounting for 11% (886 individuals) of the total abundance. In Montreal, the mean number of exotic species per site (10.16 ± 0.51) was significantly higher (*t*_1,45_ = 7.338, *P* < 0.001) than in Quebec City (5.27 ± 0.55). Their mean abundance per site was also significantly higher (*t*_1,45_ = 4.394, *P* < 0.001) in Montreal (117.88 ± 20.46) than in Quebec City (22.23 ± 4.13). Community gardens harboured significantly more exotic bees than urban parks (*F*_2,21_ = 3.75; *P* = 0.0405). No significant differences were found for their abundance (*F*_2,21_ = 2.30, *P* = 0.128) between the habitats in Quebec City.

In Montreal, the mean number of abundant species per site (27.17 ± 0.76) was also significantly higher (*t*_1,45_ = 21.30, *P* < 0.001) and their mean abundance per site (816.52 ± 148.52) was significantly greater (*t*_1,45_ = 3.99, *P* < 0.001) compared to Quebec City (10.00 ± 0.29 and 233.73 ± 32.20 respectively). When comparing the mean number of bees per pan trap in all three urban habitats (park, cemetery and community garden) in each city, the results for Montreal showed that abundance in each habitat was significantly different (*F*_2,44_ = 5.915; *p* = 0.005). The mean number of bees per pan trap in community gardens (38.96 ± 6.71) was significantly higher than in cemeteries (16.21 ± 2.50). No difference was found for parks. The same conclusion was found for the data collected from netting (*F*_2,44_ = 6.413; *p* = 0.003). For Quebec City, no significant difference (*F*_2,44_ = 1.25; *p* = 0.297) was found between the three habitats for mean number of specimens/pan trap. However, community gardens and parks showed a significantly higher abundance (*F*_2,44_ = 4.63; *p* = 0.015) than cemeteries for data collected from netting.

**Figure 2:**
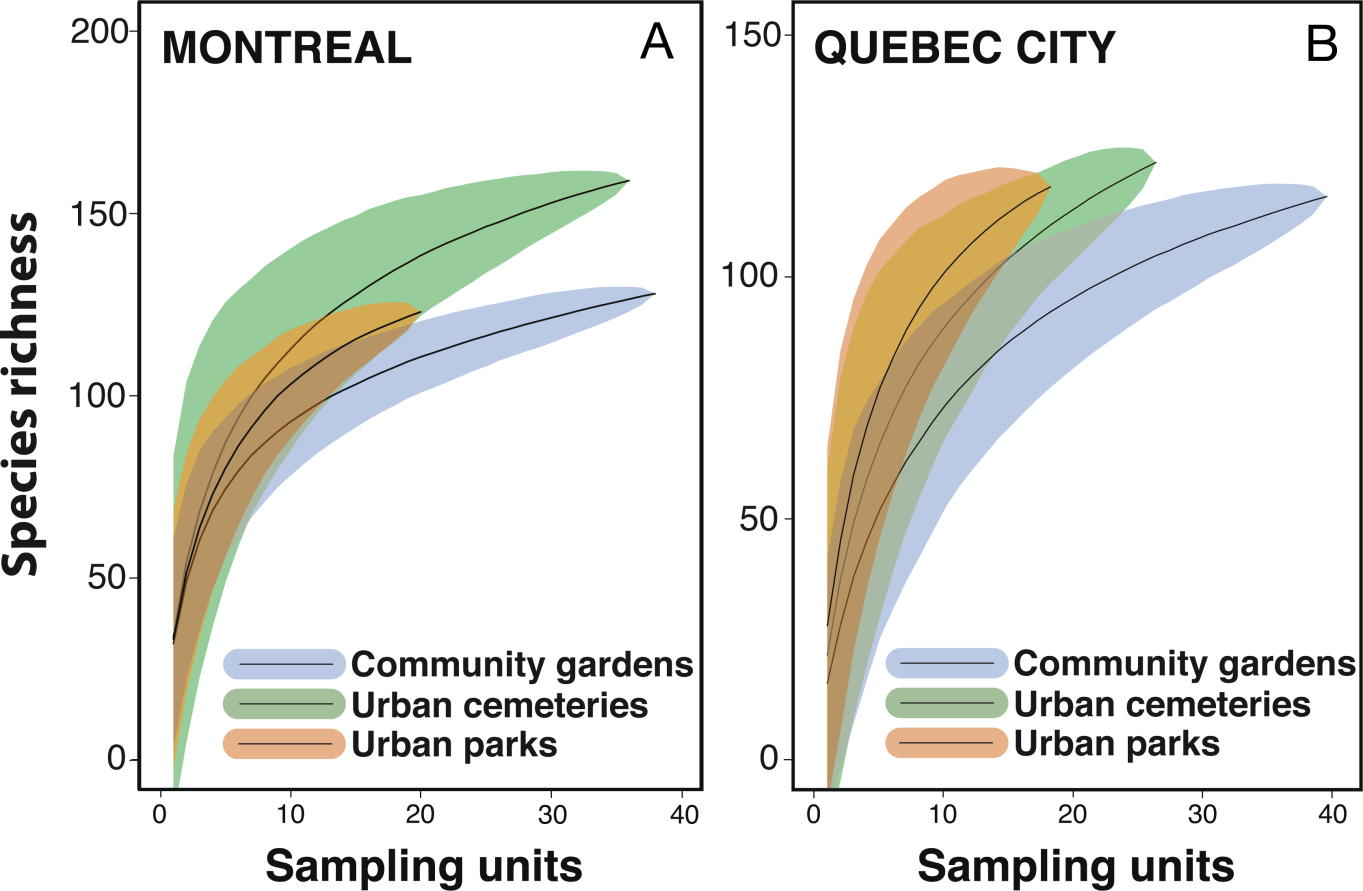
Observed species-accumulation curves (thin solid line) and their associated standard deviation (SD) for each habitat type investigated in Montreal (A) and Quebec City (B).

**Figure 3:**
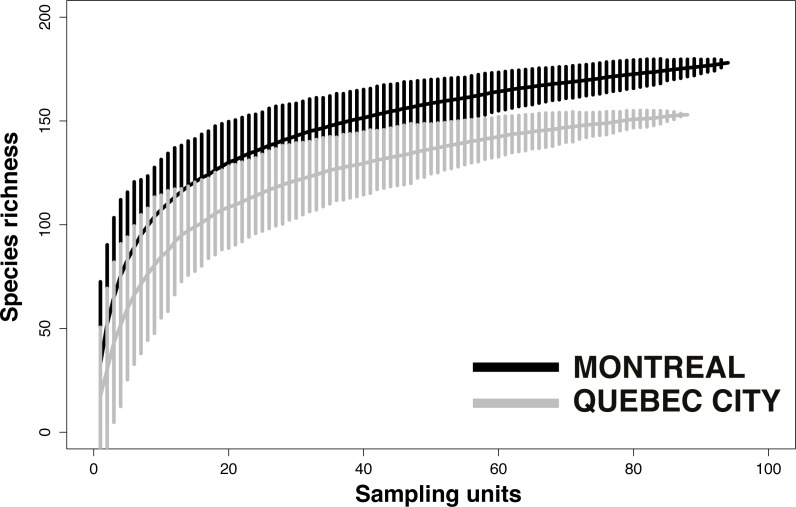
The figure shows the mean species accumulation curves and their associated standard deviation from *n* = 999 random permutations of the data.

Despite considerable sampling efforts, none of the three species’ accumulation curves for the habitats (Fig. 2) reached a plateau for either city. For Montreal, the highest richness was associated with cemeteries (157 species), followed by community gardens (128 species) and parks (124 species). For Quebec City, cemeteries hosted 122 species, parks 119 and community gardens 117. Overall, the species accumulation curves comparing the two cities (Fig. 3) reflected a good sampling effort, but curves still did not reach saturation, with 177 species for Montreal and 152 species for Quebec City. Species richness was also extrapolated with different estimators (Chao, Jackknife 1, Jackknife 2, and Bootstrap), as shown in Table 2. The highest estimation occurred with the second order estimator Jackknife, with 219.61 and 190.72 species, and the lowest occurred with Bootstrap, with 192.63 and 167.91 species for Montreal and Quebec City, respectively.

**Table 2:** Observed and extrapolated species richness (standard deviation, S.D.) between Montreal and Quebec City.

Extrapolated species richness					
City	Observed species	Chao ± SD	Jacknife 1 ± SD	Jacknife 2	Bootstrap ± SD
Montreal	177	203.73 ± 12.04	207.79 ± 7.75	219.61	192.63 ± 5.06
Quebec City	152	174.02 ± 9.71	182.67 ± 9.25	190.72	167.91 ± 5.92

**Figure 4:**
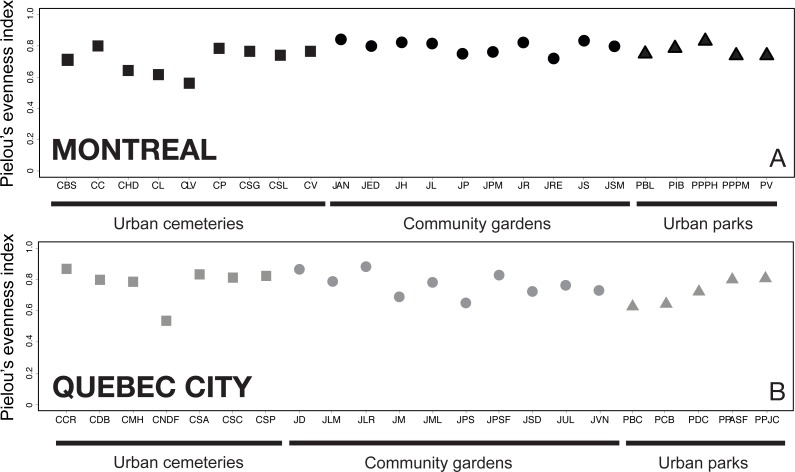
Analysis of Pielou’s *J*′ index of species evenness (Pielou, 1966) among sampling sites in Montreal (A) and Quebec City (B). The index indicates how close in number each species is in each sampling site; a value of 1 refers to a completely numerically even community of species, whereas a value closer to 0 indicates a numerically unbalanced community. The results show that, particularly in Montreal, more uneven communities are found in urban cemeteries than in community gardens or city parks.

Evenness analysis for both cities showed that for most of the sites, the evenness index (*J*′) value varies by around 0.8, which indicates good community evenness (Fig. 4). A total of eight sites showed a lower evenness value: CHD, CLV, CL, CNDF, JM, JPS, PCB and PBC (see Table 1 for details on acronyms). Species primarily responsible for the decrease in evenness of those sites are abundant species and their identity is provided via the heat map.

**Figure 5:**
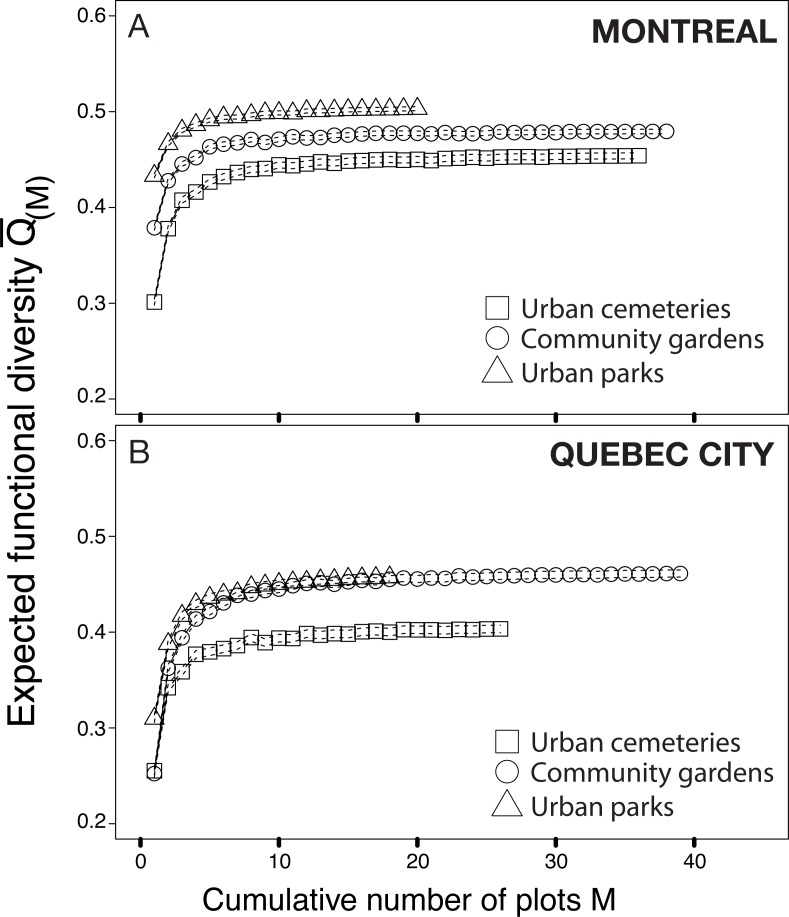
Functional rarefaction curves (mean expected functional diversity *Q*(*M*) as a function of the cumulative number of sampling plots M) for species abundance data from the three habitat types investigated in Montreal (A) and Quebec City (B). The rarefaction curves are the result of 999 randomizations and the dotted lines of each curve are bootstrapped 95% confidence intervals.

The functional rarefaction curves (Fig. 5) show the expected functional trait diversity for the three types of urban habitats. The curves increase asymptotically from their initial point and nearly reach the *Q* value (*N*) following the increase of *M* (possible combinations of sampling plots). The results indicate that urban cemeteries are significantly less functionally diverse than community gardens and urban parks irrespective of the city considered, and that urban parks harbour significantly higher functional trait diversity in each city; community gardens have significantly higher functional trait diversity than urban cemeteries and are functionally less diverse than urban parks in Montreal. These results contrast with the higher levels of species richness found in each habitat type and suggest that the higher levels of species richness found in urban cemeteries are associated to higher levels of functional trait redundancy and a lower expected functional trait diversity compared to other habitat types in both Montreal and Quebec City.

**Figure 6:**
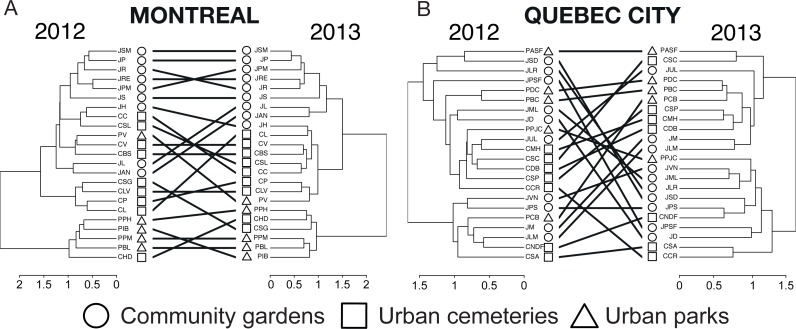
A tanglegram approach to the analysis of changes in wild bee community structure among sites and habitat types between 2012 and 2013 in Montreal (A) and Quebec City (B). Here, a hierarchical clustering with multiscale bootstrap resampling was performed using abundance data of wild bees for each city and each year on the same sampling sites. The 2012 and 2013 dendrograms of each city were aligned so as to minimize the number of crossings between inter-tree edges, and matching leaves (i.e., sampling sites) were connected by inter-tree edges. We measured the quality of the alignment of the two trees in the tanglegram layout by quantifying (i) the “entanglement” index (0, perfect match with straight horizontal edges between matching sites; 1, no match at all), (ii) Baker’s Gamma Index, a measure of statistical similarity between two dendrograms (−1, statistically highly dissimilar; 0, not statistically similar; 1, statistically similar) and (iii) the cophenetic correlation coefficient (CCC), a measure of how faithfully the 2013 dendrogram (particularly the cophenetic distances obtained) reflects the dissimilarities among sites observed in 2012 (i.e., the reference tree) (−1, the two dendrograms are statistically dissimilar; near 0 values, the two trees are not statistically similar; 1, the two dendrograms are statistically similar).

The tanglegrams (Fig. 6) illustrate community partitioning among all sites in both cities and the quality of alignment between pairs of dendrograms of 2012 and 2013. For Montreal, the presence of a nested park cluster indicates that the bee communities in parks were similar for this habitat but different from the bee communities found in community gardens and cemeteries. In Quebec City, none of the three types of urban habitats was isolated in a cluster, indicating a similarity in community composition among all habitats. The three types of metrics (entanglement, Baker’s ɣ index and cophenetic correlation coefficient) measuring the degree of congruence between the clustering analyses highlighted a better alignment for Montreal: a value of 0.128 for the entanglement is considered good, since this metric ranges from 0 to 1 with 0 being a perfect value of congruence. Baker’s ɣ index value of 0.741 and the cophenetic correlation coefficient (CCC) of 0.786, which both range from −1 to 1, with 0 meaning the two trees are not statistically similar, also demonstrate a good similarity between both trees. This suggests that Montreal’s bee communities were more stable over time than those in Quebec City, where there was a low degree of congruence between the trees, with 0.634 (entanglement), −0.009 (Baker’s index) and 0.034 (CCC).

Beta diversity partitioning was performed using two different methods, following Cardoso et al. (2014) and Baselga & Orme (2012). Species turnover is the variable of interest for this study, and is represented by *β*repl and *β*sim (Table 3). Both values showed low species turnover and nestedness for Montreal and Quebec City.

**Table 3:** Beta diversity partitioning with the Cardoso et al. (2014) method and the Baselga & Orme (2012) method.

City	Method Cardoso			Method Baselga & Orme		
	*β*total	*β*repl	*β*rich	*β*sor	*β*nes	*β*sim
Montreal	0.356	0.233	0.124	0.250	0.098	0.153
Quebec City	0.468	0.240	0.228	0.228	0.021	0.208

**Notes.**

Partitioning of beta diversity with the Cardoso method: total beta diversity (*β*total), species replacement “turnover” (*β*repl)_ and species difference (*β*rich). Partitioning of beta diversity with the Baselga & Orme method; total community dissimilarity (*β*sor, Sørensen dissimilarity), “true” species turnover (*β*sim, Simpson dissimilarity) and nestedness-resultant dissimilarity (*β*nes, nestedness). Both methods were used to analyze and compare habitat types and sites in Montreal and Quebec City.

**Figure 7:**
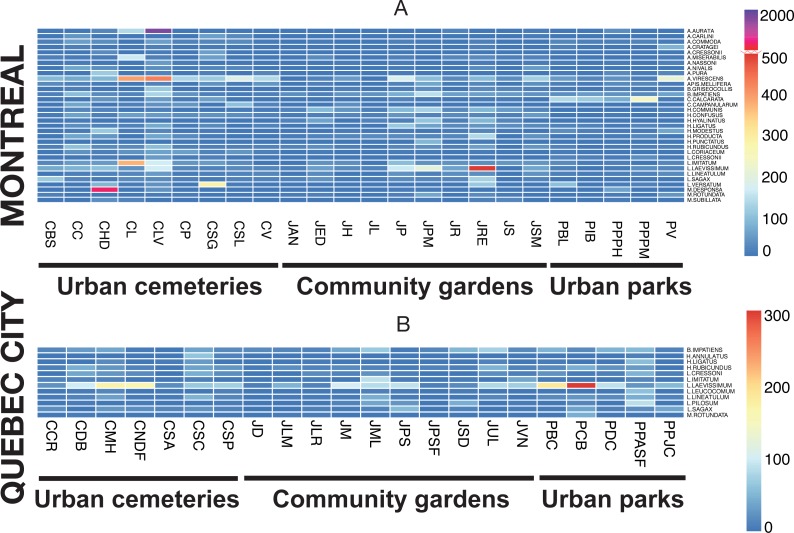
Heat map illustrating the abundant species in both cities (Montreal (A) and Quebec City(B)) and in all three urban habitats (cemeteries, community gardens and city parks).

The rank-abundance analysis did not identify dominant species (>50% individuals/per site), but did show abundant species for several sites. The heat map (Fig. 7) illustrates species abundance only for those species that numbered 50 or more individuals in one of the two sampling seasons, with the two sampling methods pooled. Two species were highly abundant, *Melissodes desponsa* in the Hawthorn-Dale cemetery (CHD) and *Augochlorella aurata* in the Lakeview cemetery (CLV), with 789 and 1,043 individuals respectively, thus decreasing the evenness value of those sites. For Lachine Cemetery (CL), *Agapostemon virescens* and *Lasioglossum imitatum* were especially abundant. For Quebec City, *Lasioglossum laevissimum* was responsible for the lower evenness values of CNDF, JM, JPS, PCB and PBC (again, please refer to Table 1 for acronyms). This species was also abundant in Montreal, especially in the Rosemont–Églantier garden (JRE). The heat map also shows which species were abundant in both urban settings, 33 species for Montreal and 12 for Quebec City, while nine (75%) were shared.

## Discussion

The main objective of our study was to compare wild bee diversity among three types of urban habitats, cemeteries, parks and community gardens, in two Canadian cities, Montreal and Quebec City. We used broad measures of community structure and diversity, such as partitioning of beta diversity, occurrence of abundant and exotic species, evenness, spatio-temporal stability and functional trait diversity. Our results show that abundant and exotic species play a central role in structuring these urban bee communities. Urban parks had the highest expected functional trait diversity of bees in both cities, closely followed by community gardens. Our work represents one of the largest bee diversity studies conducted in urban centres, particularly for Northeastern North America.

### Urban bee diversity

There are some 365 bee species in the province of Quebec (Ascher & Pickering, 2014). Our sampling protocol allowed us to capture 200 species from six bee families: Andrenidae, Apidae, Colletidae, Halictidae, Megachilidae and Melittidae. The Halictidae and the Andrenidae were the most diverse groups, with 60 and 53 species respectively, followed by the Megachilidae, with 38 species and the Apidae, with 36 species. However, the accumulation curves, which did not reach a plateau for either city or species estimators, showed that our sampling could have captured more species. Nonetheless, with 177 species found in Montreal and 152 in Quebec City, both cities host diverse wild bee communities, confirming findings of previous studies elsewhere. For example, Fortel et al. (2014) documented 291 species in Lyon, France, and 104 species were found in Poznan, Poland (Banaszak-Cibicka & Zmihorski, 2012). In North America, Fetridge, Ascher & Langellotto (2008) reported 110 species in New York’s suburbs (United-States) and Tommasi et al. (2004) found 56 species in Vancouver, Canada. These diverse communities of wild bees seem indeed to be favoured by the highly heterogeneous habitats occurring in anthropogenic environments (Winfree, Bartomeus & Cariveau, 2011). Contrasting habitats arise because the growth of urban environments creates different levels of disturbance, ranging from high intensity in the city core to low intensity at the outskirts.

Urban landscapes are also characterized by high floral diversity that provides forage for a wide variety of bees (Loram et al., 2007; Frankie et al., 2005; Gaston et al., 2005). Floral resources should then determine the quality and suitability of the different kinds of green spaces to urban bees. Relative bee abundance did not differ substantially between the three types of green spaces studied in Quebec City, a finding similar to that was reported by Baldock et al. (2015) in the United Kingdom. For Quebec City, community gardens and parks showed a higher bee abundance than cemeteries, a finding based only on specimens collected by netting. In Montreal, community gardens showed the highest relative abundance compared to cemeteries, a finding, this time, based on specimens collected by both netting and pan traps. The abundance of flowering plants and their density in community gardens likely explain our results. Similarly, Frankie et al. (2005) observed the highest bee diversity and abundance in gardens with high numbers of bee-attracting plants. Andersson, Barthel & Ahrné (2007) found that bumblebees clearly benefit from the management practices in allotment gardens, where a much higher abundance of bumblebees was observed compared to parks and cemeteries. Even if larger sites could offer more nesting opportunities and thus host a higher abundance of bees than smaller sites, we found that cemeteries, often large in size, had the lowest abundance per pan trap, and with the netting technique.

### Community structure

Cities often recruit some of the same species, which then become widespread and synanthropic (Shochat et al., 2010), but wild bees are highly mobile insects with relatively high diversity in cities (e.g., Fortel et al., 2014; Banaszak-Cibicka & Zmihorski, 2012; Bates et al., 2011; Fetridge, Ascher & Langellotto, 2008). Montreal and Quebec City shared 129 species (Fig. 1), but most are common throughout Northeastern North America. When comparing city species lists, New York’s suburban community gardens (Fetridge, Ascher & Langellotto, 2008), Toronto’s green roofs (Colla, Willis & Packer, 2009) and Guelph’s surroundings (Horn, 2010) share 64%, 87% and 69% of their species, respectively, with those found in Montreal and Quebec City combined. However, cranberry crops (A Gervais, V Fournier, CS Sheffield & M Chagnon, 2016, unpublished data) and lowbush blueberry fields (Moisan-DeSerres, Chagnon & Fournier, 2015) in the province of Quebec also share a high proportion, 48% and 63% of their species, respectively. Moreover, natural habitats surrounding cranberry crops (A Gervais, V Fournier, CS Sheffield & M Chagnon, 2016, unpublished data) and the Black Rock Forest preserve (New York State, USA) (Giles & Ascher, 2006) also share 51% and 59% of their species, respectively, with those found in our study (Table 4). These examples suggest that the habitat does not specifically define the presence of certain bee species only across cities. In an urban context, native synanthropic species (those associated with humans) may be difficult to identify because they also occur in natural habitats. Still, in our study, nine species were abundant in both Montreal and in Quebec City (see Fig. 7). Species that are more often abundant and are found in dense populations in cities can be described as synurbic (Francis & Chadwick, 2012), but other cities (Colla, Willis & Packer, 2009; Horn, 2010), suburban areas (Richards et al., 2011), crops (A Gervais, V Fournier, CS Sheffield & M Chagnon, 2016, unpublished data; Moisan-DeSerres, Chagnon & Fournier, 2015; Payette, 2013) and natural habitats (Grixti & Packer, 2006) located in the same geographic area as our two cities also showed a high variation in abundance of those nine species.

**Table 4:** Bee species collected every two weeks from May to September 2012 and 2013 in Montreal and Quebec City, with their abundance for each city, status (native or exotic), and pollen specificity. The species are sorted by phylogenetic order.

Species	Montreal	Quebec	Native or exotic	Pollen specificity
*Colletes americanus* Cresson, 1868	0	1	N	P
*Colletes simulans* Cresson, 1868	11	1	N	O
*Hylaeus (Cephalylaeus) basalis* (Smith, 1853)	0	1	N	P
*Hylaeus (Hylaeus) annulatus* (Linnaeus, 1758)	100	92	N	P
*Hylaeus (Hylaeus) communis* Nylander, 1852	633	21	E	P
*Hylaeus (Hylaeus) leptocephalus* (Morawitz, 1871 [“1870”])	41	6	E	P
*Hylaeus (Hylaeus) mesillae* (Cockerell, 1896)	201	5	N	P
*Hylaeus (Prosopis) affinis* (Smith, 1853)	142	26	N	P
*Hylaeus (Prosopis) modestus* Say, 1837	359	230	N	P
*Hylaeus (Prosopis) nelumbonis* (Robertson, 1890)	2	0	N	O
*Hylaeus (Spatulariella) hyalinatus* Smith, 1842	357	2	E	P
*Hylaeus (Spatulariella) punctatus* (Brullé, 1832)	108	0	E	P
*Macropis (Macropis) nuda* (Provancher, 1882)	0	1	N	O
*Augochloropsis (Paraugochloropsis) metallica* (Fabricius, 1793)	20	0	N	P
*Augochlorella aurata* (Smith, 1853)	2,126	61	N	P
*Augochlora (Augochlora) pura pura* (Say, 1837)	262	20	N	P
*Agapostemon (Agapostemon) texanus* Cresson, 1872	17	1	N	P
*Agapostemon (Agapostemon) virescens* (Fabricius, 1775)	2,552	1	N	P
*Sphecodes carolinus* Mitchell, 1956	1	4	N	P
*Sphecodes clematidis* Robertson, 1897	5	8	N	P
*Sphecodes confertus* Say, 1837	1	0	N	P
*Sphecodes cressonii* (Robertson, 1903)	1	0	N	P
*Sphecodes dichrous* Smith, 1853	4	3	N	P
*Sphecodes mandibularis* Cresson, 1872	0	2	N	P
*Sphecodes ranunculi* Robertson, 1897	22	27	N	P
*Sphecodes solonis* Graenicher, 1911	0	1	N	P
*Halictus (Odontalictus) ligatus* Say, 1837	605	146	N	P
*Halictus (Protohalictus) rubicundus* (Christ, 1791)	603	411	N	P
*Halictus (Seladonia) confusus confusus* Smith, 1853	558	144	N	P
*Lasioglossum (Dialictus) abanci* (Crawford, 1932)	0	1	N	P
*Lasioglossum (Dialictus) asteris* (Mitchell, 1960)	1	1	N	P
*Lasioglossum (Dialictus) cephalotes* (Dalla Torre, 1896)	1	0	N	P
*Lasioglossum (Dialictus) coeruleum* (Robertson, 1893)	26	0	N	P
*Lasioglossum (Dialictus) cressonii* (Robertson, 1890)	188	334	N	P
*Lasioglossum (Dialictus) dreisbachi* (Mitchell, 1960)	7	0	N	O
*Lasioglossum (Dialictus) ephialtum*Gibbs, 2010	306	82	N	P
*Lasioglossum (Dialictus) heterognathum* (Mitchell, 1960)	4	0	N	P
*Lasioglossum (Dialictus) hitchensi* Gibbs, 2012	64	17	N	P
*Lasioglossum (Dialictus) imitatum* (Smith, 1853)	1,221	208	N	P
*Lasioglossum (Dialictus) isawsum* Gibbs, 2011	1	0	N	P
*Lasioglossum (Dialictus) laevissimum* (Smith, 1853)	1,720	1,812	N	P
*Lasioglossum (Dialictus) leucocomum* (Lovell, 1908)	54	122	N	P
*Lasioglossum (Dialictus) lineatulum* (Crawford, 1906)	434	311	N	P
*Lasioglossum (Dialictus) michiganense* (Mitchell, 1960)	1	13	N	P
*Lasioglossum (Dialictus) nigroviride* (Graenicher, 1911)	14	35	N	P
*Lasioglossum (Dialictus) oblongum* (Lovell, 1905)	10	14	N	P
*Lasioglossum (Dialictus) paradmirandum* (Knerer & Atwood, 1966)	1	0	N	P
*Lasioglossum (Dialictus) perpunctatum* (Ellis, 1913)	0	68	N	P
*Lasioglossum (Dialictus) pilosum* (Smith, 1853)	59	248	N	P
*Lasioglossum (Dialictus) planatum* (Lovell, 1905)	11	5	N	O
*Lasioglossum (Dialictus) sagax* (Sandhouse, 1924)	554	315	N	P
*Lasioglossum (Dialictus) smilacinae* (Robertson, 1897)	2	0	N	P
*Lasioglossum (Dialictus) subversans* (Mitchell, 1960)	0	1	N	P
*Lasioglossum (Dialictus) taylorae*Gibbs, 2010	0	2	N	O
*Lasioglossum (Dialictus) tegulare* (Robertson, 1890)	149	53	N	P
*Lasioglossum (Dialictus) tenax* (Sandhouse, 1924)	2	1	N	P
*Lasioglossum (Dialictus) trigeminum* Gibbs, 2011	9	0	N	O
*Lasioglossum (Dialictus) versans* (Lovell, 1905)	99	58	N	P
*Lasioglossum (Dialictus) versatum* (Robertson, 1902)	664	20	N	P
*Lasioglossum (Dialictus) viridatum* (Lovell, 1905)	3	1	N	P
*Lasioglossum (Dialictus) zephyrum* (Smith, 1853)	18	2	N	P
*Lasioglossum (Evylaeus) cinctipes* (Provancher, 1888)	78	87	N	P
*Lasioglossum (Hemihalictus) birkmanni* (Crawford, 1906)	7	3	N	P
*Lasioglossum (Hemihalictus) foxii* (Robertson, 1895)	1	16	N	P
*Lasioglossum (Hemihalictus) macoupinense* (Robertson, 1895)	37	45	N	P
*Lasioglossum (Hemihalictus) inconditum* (Cockerell, 1916)	0	2	N	P
*Lasioglossum (Lasioglossum) athabascense* (Sandhouse, 1933)	4	0	N	P
*Lasioglossum (Lasioglossum) coriaceum* (Smith, 1853)	348	154	N	P
*Lasioglossum (Leuchalictus) zonulum* (Smith, 1848)	100	93	E	P
*Lasioglossum (Leuchalictus) leucozonium* (Schrank, 1781)	76	62	E	P
*Lasioglossum (Sphecogastra) comagenense* (Knerer & Atwood, 1964)	2	0	N	P
*Lasioglossum (Sphecogastra) quebecense* (Crawford, 1907)	13	4	N	P
*Lasioglossum (Sphecogastra) oenotherae* (Stevens, 1920)	111	1	N	O
*Andrena (Andrena) clarkella* (Kirby, 1802)	1	1	N	O
*Andrena (Andrena) frigida* Smith, 1853	3	11	N	P
*Andrena (Andrena) mandibularis* Robertson, 1892	29	1	N	P
*Andrena (Andrena) milwaukeensis* Graenicher, 1903	4	18	N	P
*Andrena (Andrena) rufosignata* Cockerell, 1902	0	4	N	P
*Andrena (Callandrena s.l) asteris* Robertson, 1891	0	1	N	O
*Andrena (Callandrena s.l) simplex* Smith, 1853	1	0	N	O
*Andrena (Cnemiandrena) chromotricha* Cockerell, 1899	15	0	N	P
*Andrena (Cnemiandrena) hirticincta* Provancher, 1888	10	17	N	O
*Andrena (Cnemiandrena) nubecula* Smith, 1853	2	17	N	O
*Andrena (Euandrena) algida* Smith, 1853	0	1	N	P
*Andrena (Euandrena) geranii* Robertson, 1891	2	10	N	O
*Andrena (Gonandrena) fragilis* Smith, 1853	1	0	N	O
*Andrena (Gonandrena) integra* Smith, 1853	4	3	N	P
*Andrena (Gonandrena) persimulata* Viereck, 1917	3	31	N	P
*Andrena (Gonandrena) platyparia* Robertson, 1895	0	2	N	P
*Andrena (Holandrena) cressonii cressonii* Robertson, 1891	101	0	N	P
*Andrena (Larandrena) miserabilis* Cresson, 1872	395	13	N	P
*Andrena (Leucandrena) erythronii* Robertson, 1891	2	0	N	O
*Andrena (Melandrena) carlini* Cockerell, 1901	153	37	N	P
*Andrena (Melandrena) commoda* Smith, 1879	355	53	N	P
*Andrena (Melandrena) dunningi* Cockerell, 1898	2	0	N	P
*Andrena (Melandrena) nivalis* Smith, 1853	284	78	N	P
*Andrena (Melandrena) regularis* Malloch, 1917	1	0	N	P
*Andrena (Melandrena) vicina* Smith, 1853	23	14	N	P
*Andrena (Micrandrena) ziziae* Robertson, 1891	2	2	N	O
*Andrena (Micrandrena) nigrae* Robertson, 1905	4	0	N	P
*Andrena (Plastandrena) crataegi* Robertson, 1893	159	15	N	P
*Andrena (Ptilandrena) distans* Provancher, 1888	1	0	N	O
*Andrena (Ptilandrena) erigeniae* Robertson, 1891	2	2	N	O
*Andrena (Rhacanrena) brevipalpis* Cockerell, 1930	1	0	N	P
*Andrena (Rhacandrena) robertsonii* Dalla Torre, 1896	6	4	N	P
*Andrena (Scrapteropsis) alleghaniensis* Viereck, 1907	0	4	N	P
*Andrena (Scrapteropsis) imitatrix* Cresson, 1872	8	11	N	P
*Andrena (Scrapteropsis) morrisonella* Viereck, 1917	20	2	N	P
*Andrena (Simandrena) nassonii* Robertson, 1895	163	53	N	P
*Andrena (Simandrena) wheeleri* Graenicher, 1904	60	76	N	P
*Andrena (Taeniandrena) wilkella* (Kirby, 1802)	60	79	E	P
*Andrena (Thysandrena) w-scripta* Viereck, 1904	8	3	N	P
*Andrena (Thysandrena) bisalicis* Viereck, 1908	2	0	N	P
*Andrena (Trachandrena) ceanothi* Viereck, 1917	3	17	N	P
*Andrena (Trachandrena) forbesii* Robertson, 1895	19	27	N	P
*Andrena (Trachandrena) hippotes* Robertson, 1895	167	33	N	P
*Andrena (Trachandrena) rugosa* Robertson, 1891	2	2	N	P
*Andrena (Trachandrena) sigmundi* Cockerell, 1902	0	5	N	O
*Andrena (Trachandrena) spiraeana* Robertson, 1895	23	7	N	O
*Andrena (Tylandrena) erythrogaster* (Ashmead, 1890)	1	0	N	O
*Andrena (Tylandrena) perplexa* Smith, 1853	1	0	N	P
*Calliopsis (Calliopsis) andreniformis* Smith, 1853	7	2	N	P
*Pseudopanurgus andrenoides* (Smith, 1853)	0	1	N	P
*Pseudopanurgus parvus* (Robertson, 1892)	5	0	N	P
*Pseudopanurgus helianthi* Mitchell, 1960	10	1	N	P
*Perdita (Perdita) octomaculata* (Say, 1824)	0	4	N	O
*Anthidium (Anthidium) manicatum* Linnaeus, 1758	86	25	E	P
*Anthidium (Anthidium) florentinum* Fabricius, 1775	147	0	E	P
*Anthidium (Proantidium) oblongatum* (Illiger, 1806)	74	0	E	P
*Heriades (Neotrypetes) carinata* Cresson, 1864	5	17	N	P
*Heriades (Neotrypetes) leavitti* Crawford, 1913	6	0	N	P
*Hoplitis (Hoplitis) anthocopoides* Schenck, 1853	1	0	E	P
*Hoplitis (Alcidamea) pilosifrons* (Cresson, 1864)	6	6	N	P
*Hoplitis (Alcidamea) producta producta* (Cresson, 1864)	587	69	N	P
*Hoplitis (Alcidamea) spoliata* (Provancher, 1888)	9	0	N	P
*Osmia (Diceratosmia) conjuncta* Cresson, 1864	13	22	N	P
*Osmia (Helicosmia) caerulescens* Linnaeus, 1758	0	2	E	P
*Osmia (Melanosmia) albiventris* Cresson, 1864	68	14	N	P
*Osmia (Melanosmia) atriventris* Cresson, 1864	21	12	N	P
*Osmia (Melanosmia) bucephala* Cresson, 1864	0	2	N	P
*Osmia (Melanosmia) pumila* Cresson, 1864	6	2	N	P
*Osmia (Melanosmia) simillima* Smith, 1853	2	3	N	P
*Osmia (Melanosmia) tersula* Cockerell, 1912	0	11	N	P
*Osmia (Osmia) lignaria* Say, 1837	9	3	N	P
*Osmia (Osmia) taurus* Smith, 1873	1	0	E	P
*Chelostoma (Foveosmia) campanularum* (Kirby, 1802)	311	0	E	P
*Chelostoma (Gyrodromella) rapunculi* (Lepeletier, 1841)	169	10	E	P
*Chelostoma (Prochelostoma) philadelphi* (Robertson, 1891)	31	0	N	P
*Megachile (Chelostomoides) campanulae* (Robertson, 1903)	23	0	N	P
*Megachile (Eutricharaea) rotundata* (Fabricius, 1787)	249	69	E	P
*Megachile (Litomegachile) brevis* Say, 1837	6	0	N	P
*Megachile (Litomegachile) mendica* Cresson, 1878	21	0	N	P
*Megachile (Litomegachile) texana* Cresson, 1878	128	24	N	P
*Megachile (Megachile) centuncularis* (Linnaeus, 1858)	46	11	E	P
*Megachile (Megachile) lapponica* Thomson, 1872	33	2	N	P
*Megachile (Megachile) inermis* Provancher, 1888	5	81	N	P
*Megachile (Megachile) relativa* Cresson, 1878	15	1	N	P
*Megachile (Xanthosarus) frigida* Smith, 1853	40	25	N	P
*Megachile (Xanthosarus) gemula* Cresson, 1878	2	0	N	P
*Megachile (Xanthosarus) latimanus* Say, 1823	14	1	N	P
*Megachile (Xanthosarus) melanophaea* Smith, 1853	1	19	N	P
*Megachile (Sayapis) pugnata* Say, 1837	1	15	N	P
*Coelioxys (Boreocoelioxys) octodentata* Say, 1824	4	0	N	P
*Coelioxys (Boreocoelioxys) porterae* Cockerell, 1900	10	12	N	P
*Xylocopa (xylocopoides) virginica* Linnaeus, 1771	1	0	N	P
*Ceratina (Zadontomerus) calcarata* Robertson, 1900	779	160	N	P
*Ceratina (Zadontomerus) dupla* Say, 1837	109	24	N	P
*Ceratina (Zadontomerus) mikmaqi* Rehan & Sheffield, 2011	102	33	N	P
*Nomada bethunei* Cockerell, 1903	48	23	N	P
*Nomada cressonii* Robertson, 1893	3	0	N	P
*Nomada denticulata* Robertson, 1902	9	2	N	P
*Nomada luteoloides* Robertson, 1895	30	9	N	P
*Nomada maculata* Cresson, 1863	12	10	N	P
*Nomada parva* Robertson, 1900	0	1	N	P
*Nomada pygmaea* Cresson, 1963	26	1	N	P
*Nomada vicina* Cresson, 1863	2	2	N	P
*Epeolus scutellaris* Say, 1824	5	0	N	P
*Triepeolus obliteratus* Graenicher, 1911	0	1	N	P
*Triepeolus remigatus* (Fabricius, 1804)	1	0	N	P
*Triepeolus pectoralis* (Robertson, 1897)	19	0	N	P
*Holcopasites calliopsidis* (Linsley, 1943)	1	0	N	P
*Mellisodes (Eumelissodes) druriella* (Kirby, 1802)	117	23	N	O
*Melissodes (Eumelissodes) subillata* LaBerge, 1961	134	19	N	P
*Melissodes (Eumelissodes) illata* Lovell & Cockerell, 1906	74	25	N	P
*Melissodes (Eumelissodes) trinodis* Robertson, 1901	20	13	N	P
*Melissodes (Heliomelissodes) desponsa* Smith, 1854	932	58	N	O
*Peponapis (Peponapis) pruinosa* (Say, 1837)	181	7	N	O
*Anthophora (Clisodon) terminalis* Cresson, 1869	23	4	N	P
*Bombus (Bombus) terricola* Kirby, 1837	6	8	N	P
*Bombus (Psithyrus) citrinus* (Smith, 1854)	47	25	N	P
*Bombus (Thoracobombus) fervidus* (Fabricius, 1798)	15	23	N	P
*Bombus (Cullumanobombus) rufocinctus* Cresson, 1863	142	140	N	P
*Bombus (Cullumanobombus) griseocollis* (DeGeer, 1773)	311	12	N	P
*Bombus (Pyrobombus) bimaculatus* Cresson, 1863	64	56	N	P
*Bombus (Pyrobombus) impatiens* Cresson, 1863	1,097	627	N	P
*Bombus (Pyrobombus) perplexus* Cresson, 1863	8	6	N	P
*Bombus (Pyrobombus) ternarius* Say, 1837	36	44	N	P
*Bombus (Pyrobombus) vagans vagans* Smith, 1854	44	33	N	P
*Bombus (Subterraneobombus) borealis* Kirby, 1837	4	3	N	P
*Apis (Apis) mellifera* Linnaeus, 1758	416	95	E	P

The presence of dominant species decreases community evenness, and this decline in evenness is a pattern observed in several studies (e.g., Shochat et al., 2010; Marzluff, Bowman & Donnelly, 2001) reflecting the redundant negative impact of urbanization on communities. For ground arthropods, evenness declines as urban exploiters increase in relative abundance and come to dominate communities (Faeth, Bang & Saari, 2011). Rank-abundance analysis did not show the presence of dominant species (defined, for the purpose of our study, as species representing more than 50% of the total abundance of bee species at a given site), but indicated many species we defined as abundant. Our results (Fig. 4) also showed that only three sites (CHD, CL and CLV) in Montreal and one site (CNDF) in Quebec City had lower evenness values (*J*). Patterns of evenness with winged insects like bees may be different, however, because their abundance can also be influenced by flower density (Westphal, Steffan-Dewenter & Tscharntke, 2003). The general increase in plant species evenness in cities (Walker et al., 2009; Grimm et al., 2008) may explain the relatively high evenness found in our study (Fig. 4). Nevertheless, abundant species may maintain high evenness values. The most striking results emerged from those abundant species (Fig. 7), which accounted for 81% (33 species) and 59% (12 species) of the abundance for Montreal and Quebec City, respectively (Table 4). Montreal had significantly more abundant species, which were also more abundant than those in Quebec City. This suggests that only a few species may represent a much higher proportion of the whole community in both cities. Being ubiquitous and generalist are the common characteristics all those species share (except *Melissodes desponsa*). Their success could be explained by a higher tolerance to fast changing pollen/nesting resources and an opportunistic nature. Indeed, even if Montreal and Quebec City have a fair amount of oligolectic (specialist) species (21 (11.8%) and 19 (12.4%), respectively) most of the species are polylectic (generalist). As urban sprawl continues, these species may gain more territory and the pressure they apply on other species through higher foraging efficiency could eventually lead to the loss of native species (Petren & Case, 1996). Competition for resources can occur in bees (Goulson & Sparrow, 2009) especially when exotic species are involved (Lye et al., 2010).

The presence and abundance of exotic species are important components of the effects of urbanization on communities (McKinney, 2002). In our study, 18 exotic species were recorded, 17 for Montreal and 13 for Quebec City, representing 14% and 11% of total abundance, respectively. Two species were newly recorded for the continent: *Anthidium florentinum* and *Hylaeus communis*. The first was largely distributed in the three different habitats we studied and outnumbered the two other species of *Anthidium* in abundance (see species list, Table 4). Montreal is likely the entry point of this species, since it has not been recorded outside the metropolitan area. Given its distribution and abundance in Montreal, *A. florentinum* may become invasive, with a high potential for expansion throughout North America, such as what has been documented with *A. manicatum* (Gibbs & Sheffield, 2009). In addition, the presence of *A. florentinum* represents an exceptional opportunity to monitor the expansion of an exotic bee species in American cities as well. The second exotic species, *H. communis*, was also largely distributed across the three habitats, but was especially abundant in community gardens (Fig. 7). The presence of preferred cultivated plants could be the driver of its high abundance. In Montreal’s community gardens, exotic members of the *Hylaeus* genus were the most abundant group compared to other exotic species, a situation also recorded in New York by Matteson, Ascher & Langellotto (2008). Interestingly, this species, *H. communis*, and four others (*Chelostoma campanularum*, *C. rapunculi*, *Hylaeus hyalinatus*, and *H. leptocephalus*) were especially low in abundance in Quebec City. Their introduction there is probably recent, due to the city’s distance from Montreal (230 km). It would be interesting to monitor any changes in community composition and dynamic in Quebec City in the coming years. The exotic species found in our study were also found in many studies conducted in urban areas in Northeastern North America (Matteson, Grace & Minor, 2013; Richards et al., 2011; Tonietto et al., 2011; Horn, 2010; Fetridge, Ascher & Langellotto, 2008; Matteson, Ascher & Langellotto, 2008). In Eastern Canada, crops and natural habitats (A Gervais, V Fournier, CS Sheffield & M Chagnon, 2016, unpublished data; Moisan-DeSerres, Chagnon & Fournier, 2015; Grixti & Packer, 2006; Sheffield, Kevan & Smith, 2003) are currently fostering no more than five exotic species. With the exception of *Andrena wilkella*, *Lasioglossum zonulum* and *L. leucozonium*, few exotic species seem to occur in non-urban habitats, such as agricultural and natural settings, while exotic cavity nesters appear to have a preference for cities. In agreement with this observation, we found only three exotic species that were ground nesting bees whereas fourteen exotics were cavity nesters. In the Northeastern United-States, exotic bee species have increased by a factor of nine during the last century (Bartomeus et al., 2013). In this context, the link between urban sprawl and exotic bee species is becoming more apparent. These species, mostly of European origin (Sheffield, Dumesh & Cheryomina, 2011), were present in all three habitats studied here, which suggests that they probably take advantage of the high occurrence of European flowering plants in cities. Their efficiency in adapting to a new environment (another continent), foraging on exotic and native flowers and finding suitable nesting sites demonstrates the high behavioural plasticity of wild bees.

Overall, the community composition, as illustrated by the tanglegrams (Fig. 6) showed a high spatial variation between habitats and sites, as well as a high temporal variation for Quebec City, an outcome that concurs with findings in agroecosystems (Russo et al., 2015) and natural habitats (Grixti & Packer, 2006). Indeed, Quebec City’s high spatio-temporal variations were shown to underlie a low stability in community composition through time. In contrast, Montreal’s entanglement metrics showed a great similarity between the two trees (Fig. 6), suggesting low spatio-temporal variations and a relatively stable community composition through time. Partitioning the beta diversity showed that species turnover was low according to both methods (Cardoso et al., 2014; Baselga & Orme, 2012), strengthening our findings. It is known that urbanization can stabilize bird communities (Suhonen et al., 2009; Devictor et al., 2007). Moreover, as proposed by Suhonen et al. (2009), generalist species that are abundant may reduce variation in temporal stability. Such species may be well adapted to the urban environment and its resource productivity, and when abundant to the point that they outnumber other species, are more likely to be found on the same site year after year. Accordingly, Montreal was found to have more abundant species than Quebec City. Thus, we speculate that these species may contribute to the alignment of the dendrograms (Fig. 6) and that the stable bee community is the product of the abundance of those species. This in turn strongly suggests that the urban environment indeed has an impact on bee community stability and profound effects on community dynamics.

### Functional trait diversity among habitats

The results on bee trait diversity showed that urban parks, mostly composed of natural habitats, have the highest expected functional trait diversity in both cities, which has also been found when comparing natural habitats with farmlands (Forrest et al., 2015). Hierarchical clustering analysis (Fig. 6) also separated urban parks in a nested cluster in Montreal, thus indicating the presence of a different wild bee community. In contrast, the lowest functional trait diversity was attributed to cemeteries, even though we found relatively high richness (see Venn diagrams, Fig. 1) when sampling this type of habitat. This may suggest that numerous species present in cemeteries have similar traits (several species were *Lasioglossum (Dialictus)*). Thus, although the large size and vegetation of cemeteries are beneficial to biodiversity (Beninde, Veith & Hochkirch, 2015), these attributes may still not be sufficient to foster higher functional trait diversity.

In contrast, community gardens are managed sites with managed vegetation, and represent the smallest habitat studied (see Table 1). However, they successfully attracted the second best expected functional trait diversity in Montreal, and matched the urban parks curve in Quebec City. This suggests that urban agriculture in community gardens attracts bees with a wide variety of traits. The greater diversity of functional traits can be explained by an increase in resource-use efficiency in a heterogeneous environment (Díaz, Noy-Meir & Cabido, 2001). In this case, the resources are nectar and pollen, and species-specific traits influencing pollination can improve pollination services through resource-use complementarity (Martins, Gonzalez & Lechowicz, 2015). Although our results on functional trait diversity and the link with resource-use complementarity are only partial, they can serve as proxy for functional diversity in the context of pollination of wild and cultivated plants. In this framework, community gardens may contribute greatly to urban pollination services through the spillover of their high functional trait diversity. Community gardens may also produce a higher functional stability through time because multiple functional traits can buffer ecosystems against abiotic variation (Walker, Kinzig & Langridge, 1999). Thus, functional stability, as a measure of functional trait diversity, offers additional arguments regarding the potential of community gardens for the conservation of urban wild bees. The potential for conserving and enhancing bee diversity in urban settings distinguishes community gardens from urban parks and cemeteries. Indeed, the size of community gardens improves their potential for integration as a green space in urban planning strategies and programs (Lin, Philpott & Jha, 2015). Urban parks are often larger by comparison, and require deployment of numerous varied municipal human resources. Consequently, their number rarely increases in today’s cities. Additionally, city dwellers are often more inclined to exercise wildlife-friendly management and agricultural practices in the context of community gardens. By targeting specific plants, which can sometimes be attractive horticultural varieties, habitat devoted to gardening can predictably increase wild bee diversity and abundance (Garbuzov & Ratnieks, 2014; Frankie et al., 2009). Accordingly, our results showed a higher relative abundance of bees in community gardens. Creating more community gardens in urban centres, and hence more opportunities for urban agriculture to flourish, could promote and ensure the conservation of urban wild bee diversity. Indeed, when diverse floral resources are available throughout the season, urban gardens contribute to the conservation of diverse, seasonal bee taxa (Wojcik et al., 2008). Urban community gardens may also be the greatest source of potentially invasive alien plants (Smith et al., 2006). Alien species of plants can facilitate the invasion of exotic bees in disturbed habitats (Morales & Aizen, 2002), because exotic plants may be more attractive to exotic bees (Maclvor, Ruttan & Salehi, 2014).

## Conclusion

Overall, our study demonstrates that cities are characterized by a high diversity of wild bees but also by abundant, ubiquitous and exotic species, revealing the tolerance and opportunistic nature of urban bees. In general, the species assemblages did not show a specific association with habitats (except Montreal’s urban parks) and showed a high temporal and spatial variation in Quebec City. We found a high spatio-temporal stability in Montreal, which is explained by the numerous abundant species. Compared to parks, community gardens hosted more exotic bees but both type of habitats had a similar abundance per pan trap; the gardens had a much higher ratio (abundance per pan trap) than cemeteries. Urban agriculture has been shown to generate habitats with high functional trait diversity and tremendous potential for local bee conservation, while also contributing to the urban pollination service and a functionally diverse bee community. The functional trait diversity measure we established therefore gives clearer insight into the response of bees to the three types of green spaces studied. In general, even with a dataset as extensive as ours, the response of bees to several environmental factors is still affected by unexplained variation, and even more intensive sampling would be required. Yet, the use of broad measures of community structure and diversity, as well as functional trait diversity, provided important insights into wild bee communities in urban settings and sets the stage for future work on essential insects living in our cities. We have established a benchmark of the biodiversity of bees in two major cities in Eastern Canada, and suggested opportunities to further develop conservation strategies for bee species and the services they provide.

##  Supplemental Information

10.7717/peerj.3051/supp-1 Supplemental Information 1 Wild bee traits data base

10.7717/peerj.3051/supp-2 Supplemental Information 2 Raw data of wild bee occurence and species in Montreal and Quebec

## Additional Information and Declarations

### Field Study Permissions

The following information was supplied relating to field study approvals (i.e., approving body and any reference numbers): We received permission to collect insects in Montreal parks from the management of Montreal’s large parks and from the director of the Boucherville Islands National Park. Authorizations to collect at the other sites (community gardens, cemeteries and Quebec city’s parks) were given verbally.

### Data Availability

The following information was supplied regarding data availability: The raw data has been supplied as a Supplementary File.
